# TeamMate: A Longitudinal Study of New Zealand Working Farm Dogs. II. Occurrence of Musculoskeletal Abnormalities

**DOI:** 10.3389/fvets.2020.00624

**Published:** 2020-10-16

**Authors:** Katja E. Isaksen, Lori Linney, Helen Williamson, Nick J. Cave, Elizabeth J. Norman, Naomi Cogger

**Affiliations:** ^1^School of Veterinary Science, Massey University, Palmerston North, New Zealand; ^2^Vetlife, Timaru, New Zealand; ^3^College of Sciences, Massey University, Palmerston North, New Zealand

**Keywords:** musculoskeletal, longitudinal, TeamMate, incidence, working dogs, herding dogs, working farm dogs

## Abstract

Musculoskeletal injury and disease are common in dogs, and a major cause of retirement in working dogs. Many livestock farmers rely on dogs for the effective running of their farms. However, the incidence of musculoskeletal disease has not been explored in working farm dogs. Here we explore the occurrence of musculoskeletal abnormalities in 323 working farm dogs that were enrolled in TeamMate, a longitudinal study of working farm dogs in New Zealand. All dogs were free of musculoskeletal abnormalities on enrolment to the study and were present for at least one follow-up examination. During the follow-up period, 184 dogs (57%, 95% confidence interval (CI) = 52%−62%) developed at least one musculoskeletal abnormality during 4,508 dog-months at risk, corresponding to 4.1 dogs (95% CI = 3.5–4.7) with recorded abnormalities per 100 dog-months at risk. The most common abnormalities were reduced range of motion and swelling of the carpus or stifle, while the hip was the most common site of pain. No major differences in incidence rate (IR) between sexes or types of dogs were observed, though Huntaways had a slightly lower rate of carpal abnormalities than Heading dogs (IR ratio = 0.6, 95% CI = 0.3–1.0). Eighty-one of 119 dogs (68%, 95% CI = 60%−76%) that had a first musculoskeletal abormality developed a second abnormality. The most common type of abnormality that was seen in the same dog more than once was reduced range of motion in the carpus (14 of 119 dogs, 12%, 95% CI = 6%−18%). Although we do not provide data on diagnoses, the high incidence rate of recorded musculoskeletal abnormalities and dogs' high activity mean it is likely that working farm dogs are at a high risk of conditions that could impair their welfare and reduce the lengths of their working careers. Preventing and managing musculoskeletal injury and illness should be a priority for owners and veterinarians caring for working farm dogs.

## Introduction

Musculoskeletal injury and disease is common in many populations of dogs, humans, and other species (1–4) and can be a serious problem that affects overall health, welfare, and working performance (5–7). In the United Kingdom, the second most commonly recorded cause of death of dogs attending clinical practice was musculoskeletal disorders (4). In New Zealand police dogs, and United Kingdom guide dogs, the most common cause for early retirement was an inability to continue working due to musculoskeletal disease or injury (8, 9). In United States military working dogs, the most commonly recorded cause of dogs dying was degenerative joint disease (10).

Working farm dogs in New Zealand have been found to have a high prevalence of musculoskeletal disease and injury, with over 40% having at least one musculoskeletal abnormality on physical examination (11). Additionally, during a 12-month period, 14% of working farm dogs had a non-traumatic musculoskeletal health event and 12% had a traumatic musculoskeletal health event, according to owners (12). Musculoskeletal disease can be a major cause of reduced quality of life due to its potential to cause pain and limit mobility (3, 13). High levels of activity such as those seen in working farm dogs (14) can contribute to increased levels of musculoskeletal disease, limiting the dogs' ability to work. Given the reliance of New Zealand farmers on their dogs for the efficient running of their farms (15), and the economic value stock-herding dogs bring to their owners (16), high incidences of musculoskeletal injury and disease may represent a major economic cost to owners of working farm dogs. Determining what types of musculoskeletal abnormalities are the most common and whether certain dogs are at increased risk of developing musculoskeletal disease could enable veterinarians and dog owners to target preventative measures more accurately. In turn, such targeting would improve dogs' health and welfare and ensure that they stay disease-free and able to work for as long as possible.

To date, the incidence of musculoskeletal injury and disease in working farm dogs has not been investigated. The aim of this study was to describe the incidence of different types of musculoskeletal abnormalities recorded in a population of working farm dogs. We anticipated that the incidence of musculoskeletal abnormalities would be associated with the sex and type of the dogs. The incidence of dogs developing musculoskeletal abnormalities is presented, stratified by the types and locations of the abnormalities seen.

## Methods

### Study Design

TeamMate is a longitudinal study focusing on working farm dogs on the South Island of New Zealand. A companion research article describes the study design and data collection procedure in detail and presents data collected on the dogs' enrolment to the study (11). To summarize, 641 working farm dogs were convenience-sampled and enrolled in a four-year longitudinal study. All working farm dogs belonging to participating dog owners were enrolled, if they were least 18 months old and working with livestock regularly. In the current study, we included 323 dogs that did not have a recorded musculoskeletal abnormality on enrolment and that were present for at least one subsequent clinical examination.

**Figure 1:**
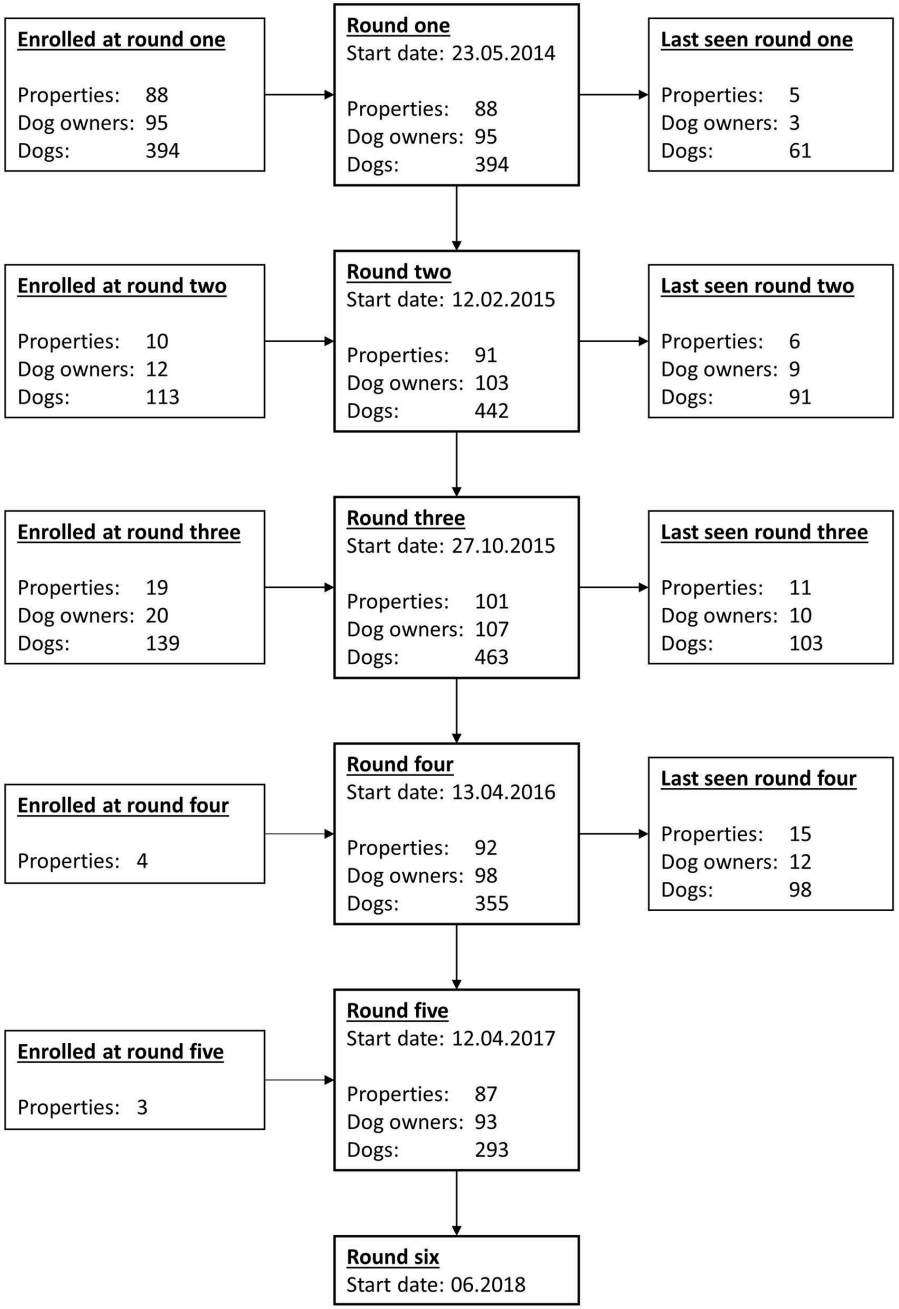
Flow chart showing the start dates of each data collection round as well as the number of farms, dog owners and dogs enrolled in TeamMate up to and including the fifth round of farm visits. Additionally, 14 properties, 16 dog owners and 68 dogs missed at least one round of data collection. Note that data for the sixth data collection round was not yet available at the time of writing. This figure was previously published by the authors (11) and is licensed for re-use under the Creative Commons Attribution 4.0 International licence.

Data collection was begun in May 2014. Data was collected approximately every eight to nine months subsequently, and data from five data collection rounds were included in the current study. The fifth data collection round was completed in November 2017. Figure 1 is a flowchart showing the start dates for each data collection round and how many dogs, owners, and farming properties were enrolled at each round. At each data collection round, farm dog owners were visited on the farm where they worked, new dog owners and dogs were enrolled, and data was collected. New dog owners and dogs were enrolled up to and including the third data collection round. New dogs included dogs belonging to previously enrolled owners that had been acquired or had aged into the study between farm visits. Some new properties were registered subsequently to the third data collection round due to participating dog owners moving or changing jobs.

At each farm visit, including on enrolment, all enrolled dogs were physically examined by veterinarians and dog owners were interviewed to collect information about the dogs' husbandry, feeding, and work. Scribes were responsible for filling in the questionnaires and taking note of any clinical findings. The physical examination included manipulation of all the major joints and encouraging the dogs to trot for a short distance to check for lameness. All physical abnormalities were recorded, irrespective of their clinical significance. All questionnaires that were used as part of the study are available as supplementary materials to a companion research article (11).

All veterinarians and scribes were trained to ensure data collection was performed in a standardized way, with veterinarians asked to record specific clinical signs rather than make general diagnoses. Training included a run-through of all questionnaires and how they should be completed as well as practical sessions that involved filling in the questionnaires and examining, scoring, and measuring farm dogs. During training sessions, normal range of motion at each joint was demonstrated in healthy working farm dogs.

Abnormalities noted on clinical examination were systematically categorized using alphanumeric codes based on the examining veterinarian's notes. Each code consisted of a letter signifying the body system involved and up to five numbers signifying the location, symmetry, type, and cause of the abnormality. Abnormalities were not mutually exclusive, and dogs could have multiple recorded abnormalities, also in the same anatomical location. Coding was carried out by a single veterinarian (LL) and checked by another person with training in veterinary health. Codes that were unclear or incomplete were rechecked by a veterinarian (LL and/or NJC). The complete system used for alphanumeric coding of physical abnormalities is available as supplementary materials to a companion research article (11).

Dogs that were enrolled in TeamMate, were free of recorded musculoskeletal abnormalities at enrolment, and were present for at least one follow-up clinical examination were included in the current study. Data relating to the occurrence of musculoskeletal abnormalities in these dogs are presented.

### Statistical Analysis

Abnormalities noted on physical examination were categorized according to type and location on the body. Anatomical locations and types of abnormalities were included in further data analysis if they were seen in 10% of dogs or more either on enrolment (11) or as a first musculoskeletal abnormality following enrolment. The anatomical locations included the carpals, hips, digits, and stifles, and abnormalities were categorized as “abnormal range of motion,” “hard swelling,” “painful,” “crepitus,” or “other.” Lameness on trot was recorded in 12% of dogs on enrolment (11). However, we did not include lameness in this study as we cannot know that the underlying cause of lameness is musculoskeletal. For example, dogs may be lame due to injuries to the footpads.

Dogs were classified by type based on the description provided by the owner. The most common types of dogs were Heading dogs and Huntaways, with other types of dogs combined and classified as “other.” A more detailed discussion on the classification of dog types and the differences between them can be found in a companion research paper (11). In short, Heading dogs and Huntaways are unique to New Zealand and have been bred to carry out different types of stock work. Heading dogs are similar to short-haired Border collies and weigh about 20 kg on average. They are usually trained to head and catch, and sometimes to do yard work (see Table 1 for descriptions of work types). Huntaways are larger than Heading dogs at an average weight of around 30 kg. Huntaways are usually trained to hunt and to do yard work.

**Table 1:** Modes of work done by working farm dogs in New Zealand.

**Mode of work**	**Description**
Head	The dog circles around to the head of the herd and uses its positioning to gather, stop, and redirect animals.
Hunt	The dog uses its bark and position to apply pressure on the herd from behind in order to move the animals forward.
Yard work	Any work done in stockyards and runs.
Catch	Separating one or several specific animals from the herd.

Time at risk to a first recorded musculoskeletal abnormality was calculated using an approximate calculation adapted from that described in Dohoo et al. (17), with dogs considered as having been withdrawn if they were lost to follow-up for any reason at an earlier date than their owner. The start time at risk was defined as the date on which an individual dog was enrolled in the study. Dogs were considered as no longer being at risk if they were recorded as having a musculoskeletal abnormality, if they or their owner were lost to follow-up for any reason, or if they reached the end of the study. Dogs that were recorded as having a musculoskeletal abnormality or were withdrawn were considered as being at risk until the halfway point between the date of their previous examination and the date on which the abnormality or the withdrawal was recorded. Dogs that were not recorded as having any musculoskeletal abnormalities or having been withdrawn were considered as being at risk until the date of their last recorded examination. Time at risk to a second recorded musculoskeletal abnormality was calculated in the same way as the first, except that the start time was considered as being the date on which dogs' first musculoskeletal abnormality was recorded.

Incidence rate was calculated as the number of dogs that had at least one musculoskeletal abnormality divided by the number of dog-months at risk. Note that this is not same as the number of injuries per time period. Dogs may have had more than one recorded abnormality on the same examination. Additionally, single cases of injury or disease were often coded more than once as a reflection of multiple clinical signs. For example, a dog may have swelling, reduced range of motion, and pain in the same joint. For these reasons, the number of dogs rather than the number of abnormalities were counted.

Incidence rates and 95% CIs were calculated for the first instance of any musculoskeletal abnormality in each dog as well as for the most common types and locations of abnormalities. Specific incidence rates, stratified by sex and type of dog, were calculated for each of the most common joint locations, and incidence rate ratios for sex and dog types were calculated with 95% CIs.

Incidence rate was also reported for second occurrences of musculoskeletal abnormalities. The calculation of time at risk included dogs that were recorded as having a first musculoskeletal abnormality and that were present for at least one subsequent examination. The types of abnormalities that were most commonly observed more than once in the same dog are reported.

All data analysis was done using R version 3.6.x (18).

## Results

Three hundred twenty-three dogs, belonging to 113 dog owners, did not have a recorded musculoskeletal abnormality on enrolment to TeamMate and were present for at least one follow-up clinical examination. These 323 dogs contributed 4,508 dog-months at risk. Table 2 shows the distribution of dogs by sex, age group at enrolment, type of dog. The median age at enrolment for both sexes was 3 years (IQR = 2–5 years). The median age at enrolment was 3 years (IQR = 2–4 years) for Heading dogs, 3 years (IQR = 2–5 years) for Huntaways, and 4 years (IQR = 3–8 years) for other types of dogs. For comparison, the median age on enrolment of all 641 dogs enrolled in TeamMate was 4 years (IQR = 2–6) (11).

**Table 2:** Population features of the 323 dogs enrolled in TeamMate that did not have a recorded abnormality on enrolment and were present for at least one follow-up examination.

**Variables**		**Number of dogs**	**% (95% CI)**
Sex	Female	151	47 (41–52)
	Male	172	53 (48–59)
Age on enrolment	1.5 to 2.9 years	187	58 (53–63)
	3 to 4.9 years	87	27 (22–32)
	5 to 6.9 years	27	8 (5–11)
	7 to 9.9 years	21	7 (4–9)
	10 years and above	1	0 (0–1)
Type of dog	Heading dog	165	51 (46–57)
	Huntaway	148	46 (40–51)
	Other	10	3 (1–5)

Of 323 dogs, 184 (57%, 95% CI = 52–62%) developed at least one musculoskeletal abnormality during 4,508 dog-months at risk, corresponding to 4.1 dogs (95% CI = 3.5–4.7) per 100 dog-months at risk. Table 3 describes the incidence rate of dogs' first recorded musculoskeletal abnormalities following enrolment, stratified by anatomical location and type of abnormality. Tables 4, 5 describe the distribution of incidence rates and rate ratios of the first occurrence of musculoskeletal abnormalities in the most commonly recorded anatomical locations, stratified by sex and type of dog, respectively.

**Table 3:** Number of affected dogs, incidence rate, and incidence rate ratio (with 95% CI) of first recorded musculoskeletal abnormalities stratified by the location on the body and type of the first recorded abnormality.

**Location**	**Type of abnormality**	**Number of dogs**	**IR/100 dog-months (95% CI)**	
Carpus	Abnormal range of motion*	44	1.0 (0.7–1.3)
	Painful	6	0.1 (0.1–0.3)
	Hard swelling	9	0.2 (0.1–0.4)
	Crepitus	4	0.1 (0.0–0.2)
	All carpus	53	1.2 (0.9–1.5)
Hip	Abnormal range of motion*	22	0.5 (0.3–0.7)
	Painful	18	0.4 (0.3–0.6)
	Crepitus	2	0.0 (0.0–0.2)
	Other	2	0.0 (0.0–0.2)
	All hip	39	0.9 (0.6–1.2)
Digits	Abnormal range of motion*	11	0.2 (0.1–0.4)
	Hard swelling	5	0.1 (0.0–0.3)
	Painful	24	0.5 (0.4–0.8)
	Crepitus	5	0.1 (0.0–0.3)
	All digits	36	0.8 (0.6–1.1)
Stifle	Abnormal range of motion*	7	0.2 (0.1–0.3)
	Hard swelling	4	0.1 (0.0–0.2)
	Painful	9	0.2 (0.1–0.4)
	Crepitus	9	0.2 (0.1–0.4)
	All stifle	25	0.6 (0.4–0.8)
Other	Abnormal range of motion*	41	0.9 (0.7–1.2)
	Hard swelling	30	0.7 (0.5–1.0)
	Painful	11	0.2 (0.1–0.4)
	Crepitus	6	0.1 (0.1–0.3)
	Other	8	0.2 (0.1–0.4)
	All other	86	1.9 (1.5–2.4)
All abnormalities	Abnormal range of motion*	102	2.3 (1.9–2.7)
	Hard swelling	56	1.2 (1.0–1.6)
	Painful	48	1.1 (0.8–1.4)
	Crepitus	21	0.5 (0.3–0.7)
	Other	17	0.4 (0.2–0.6)
	All abnormalities	184	4.1 (3.5–4.7)

*Data from 323 dogs that contributed 4,508 dog-months at risk. Note that many dogs were recorded as having more than one abnormality on the same examination. Anatomical locations and types of abnormalities were classed as “Other” if they were recorded in fewer than 10% of dogs on enrolment, or as a first musculoskeletal abnormality following enrolment*.

* *Two dogs were found to have abnormally increased range of motion, one in the shoulder and the other in the tarsus. The remainder had reduced range of motion*.

**Table 4:** Number of affected dogs, incidence rate, and incidence rate ratio (with 95% CI) of first recorded musculoskeletal abnormalities in a range of anatomical locations, stratified by sex.

**Location**	**Sex**	**Number of dogs**	**IR/100 dog-months (95% CI)**	**IR ratio (95% CI)**
Carpus	Female	24	1.1 (0.9–1.3)	
	Male	29	1.3 (1.1–1.5)	1.2 (0.7–2.0)
Hip	Female	25	1.1 (1.0–1.3)	
	Male	14	0.6 (0.5–0.7)	0.6 (0.3–1.1)
Digits	Female	14	0.6 (0.5–0.7)	
	Male	22	1.0 (0.8–1.1)	1.5 (0.8–3.0)
Stifle	Female	11	0.5 (0.4–0.6)	
	Male	14	0.6 (0.5–0.7)	1.3 (0.6–2.8)
Other	Female	55	1.7 (1.5–2.0)	
	Male	65	2.1 (1.8–2.4)	1.2 (0.8–1.8)
All locations	Female	86	3.8 (3.3–4.5)	
	Male	98	4.3 (3.7–5.0)	1.1 (0.8–1.5)

*One hundred fifty-one female dogs contributed 2,238 dog-months at risk and 172 male dogs contributed 2,270 dog-months at risk*.

**Table 5:** Number of affected dogs, incidence rate and incidence rate ratio (with 95% CI) of first recorded musculoskeletal abnormalities in a range of anatomical locations, stratified by type of dogs.

**Location**	**Type of dog**	**Number of dogs**	**IR / 100 dog-months (95% CI)**	**IR ratio (95% CI)**
Carpus	Heading dog	33	1.4 (1.2–1.6)	
	Huntaway	15	0.8 (0.6–0.9)	0.6 (0.3–1.0)
	Other	5	3.2 (1.8–5.9)	2.3 (0.9–6.0)
Hip	Heading dog	20	0.8 (0.7–1.0)	
	Huntaway	18	0.9 (0.8–1.1)	1.1 (0.6–2.1)
	Other	1	0.6 (0.3–1.2)	0.8 (0.1–5.7)
Digits	Heading dog	20	0.8 (0.7–1.0)	
	Huntaway	15	0.8 (0.6–0.9)	0.9 (0.5–1.8)
	Other	1	0.6 (0.3–1.2)	0.8 (0.1–5.7)
Stifle	Heading dog	14	0.6 (0.5–0.7)	
	Huntaway	9	0.5 (0.4–0.5)	0.8 (0.3–1.8)
	Other	2	1.3 (0.7–2.4)	2.2 (0.5–9.6)
Other	Heading dog	45	1.9 (1.6–2.2)	
	Huntaway	38	1.9 (1.6–2.3)	1.0 (0.7–1.6)
	Other	3	1.9 (1.0–3.6)	1.0 (0.3–3.3)
All locations	Heading dog	92	3.9 (3.3–4.5)	
	Huntaway	85	4.3 (3.7–5.1)	1.1 (0.8–1.5)
	Other	7	4.5 (2.5–8.3)	1.2 (0.5–2.5)

*One hundred sixty-five Heading dogs contributed 2,385 dog-months at risk, 148 Huntaways contributed 1,968 dog-months at risk, and 10 other types of dogs contributed 155 dog-months at risk*.

Of 184 dogs that were recorded to have had a first musculoskeletal abnormality 119 dogs (65%, 95% CI = 65%−72%) were present for at least one subsequent follow-up physical examination and contributed 1,144 dog-months at risk. Eighty-one of 119 dogs (68%, 95% CI = 60%−76%) were found to have a second musculoskeletal abnormality of any type. This corresponds to 7.1 dogs (95% CI = 5.7–8.7) per 100 dog-months at risk. Thirty-one of 119 dogs (26%, 95% CI = 18%−34%) were found to have a musculoskeletal abnormality of both the same type and in the same location on a subsequent examination. The most common abnormalities that were seen in the same dog repeatedly were reduced range of motion in the carpus (14 of 119 dogs, 12%, 95% CI = 6%−18%) and hard swelling in one or more digits (4 of 119 dogs, 3%, 95% CI = 0%−7%). All other types of abnormalities were seen repeatedly in three dogs or fewer.

## Discussion

This study confirms that musculoskeletal abnormalities are common in working farm dogs, with almost six in 10 dogs developing at least one musculoskeletal abnormality during the course of the study, at a rate of more than 4 dogs per 100 dog-months at risk. To our knowledge, this is the first time incidence rate of musculoskeletal disease or injury has been reported in a population of working dogs. Musculoskeletal disease and injury cause discomfort, pain, and loss of mobility that can have implications for the welfare of the affected dogs and is likely to cause a reduction in working capacity. In the short term, this loss of working capacity might put extra strain on the remaining dogs on farm as they are required to fill the gap or cause productivity issues on farm as the dog owner is unable to move stock efficiently. Additionally, injured dogs may have incomplete recoveries or lowered fitness following rest, while the remaining healthy dogs are given increased workloads. In humans, previous injury, lowered fitness, and overuse are all linked to further injuries such as tendinopathy, stress fractures, and osteochondrosis (19), while a survey of sled racing dogs suggested that overuse may have been the cause of certain injuries (20). In the long term, overuse and repeated injuries are risk factors for the development of chronic musculoskeletal disease such as osteoarthritis (21).

In this study, more than two thirds of dogs that had a musculoskeletal abnormality and were present for a subsequent examination were recorded to have a second musculoskeletal abnormality on a later examination, and more than a quarter were recorded as having the same abnormality a second time. The data recorded for this study focused on clinical signs rather than diagnosis, and there is no data available on whether repeated observations of abnormalities represent persistent musculoskeletal disease or new injuries in the same location. Either case, however, may be associated with the presence of chronic disease because repeated injuries may lead to chronic conditions such as osteoarthritis (21).

The carpal joint had the highest incidence rate of abnormalities in this study, and most of these involved reduced range of motion (Table 3). This type of abnormality was also, by far, the most common type to be recorded more than once in the same dogs, indicating that this type of abnormality may be more likely to persist over time than other types of abnormalities. However, more detailed data is needed to confirm or negate this assumption. Carpal injuries have been found to be common in racing Greyhounds (22, 23), while a survey of sled racing dogs suggested that carpal injuries may have been the result of overuse (20). Similarly, high activity levels may predispose working farm dogs to carpal injuries. This would explain the high incidence of carpal abnormalities seen in this study. Carpal abnormalities reported in this study rarely involved pain on manipulation, and it is likely that many were the results of minor injuries or changes caused by healing after injury. Dog owners may not consider these injuries serious enough to warrant a visit to a veterinary clinic. Given the effect of chronic musculoskeletal illness on other working dog populations, more research is warranted to quantify the effect of carpal injuries on the health and welfare of working farm dogs. Based on current data, it might be prudent for veterinarians and working dog owners to follow up dogs with carpal injuries and give them the necessary support to prevent and, if necessary, manage chronic musculoskeletal illness.

Except for a slightly higher rate of carpal abnormalities in Heading dogs than Huntaways, no major differences were seen in the rates of musculoskeletal abnormalities between the sexes or types of dogs (Tables 4, 5). The 95% CIs of the incidence rate ratios were narrow, indicating that our results are probably quite close to the “true” values in the study population. If this is accurate, any differences in the rates of musculoskeletal illness or injuries between sexes or types of working farm dogs are so small that they can probably be disregarded in clinical settings. As the occurrence of musculoskeletal disease and injury is known to increase with age (3, 24), a possible source of confounding in our results would be if there were pronounced age differences between the sexes or types of dogs. However, age differences between groups were not observed in this population. The small difference seen in the rate of carpal abnormalities could be spurious, or it could be explained by several factors. Heading dogs and Huntaways are phenotypically distinct (Isaksen et al., unpublished data), with Huntaways being on average ~10 kg heavier than Heading dogs (11). Health differences between breeds and phenotypes are commonly seen in dogs (2, 4, 25, 26). However, Heading dogs and Huntaways also do different types of stock work (11), which may put them at risk of different types of injuries. Cave et al. reported that along with automotive accidents, stock-related trauma was a major cause of injury in working farm dogs, and that Heading dogs were over-represented in comparison to Huntaways (27). Our data suggests that Heading dogs may be at slightly higher risk of carpal injuries than Huntaways, though further investigation of risk factors related to phenotypes and work in working farm dogs is needed. With carpal abnormalities being the most commonly reported in the population overall, these types of injuries should not be discounted in Huntaways based on the weak difference reported in this study.

No difference in the rate of hip abnormalities was seen between Heading dogs and Huntaways, and the overall incidence rate was around one per 100 dog-months. The majority of recorded hip abnormalities involved reduced range of motion and/or signs on pain, potentially impairing dogs' mobility, and overall welfare. A previous study by Hughes (28) suggested an 18% prevalence of hip dysplasia in working farm dogs, with Huntaways having a five times higher prevalence than Heading dogs. However, Hughes reports that the majority of dog owners had not noticed lameness in dogs that were scored as having hip dysplasia. Cave et al. (27) suggested that more Huntaways have hip dysplasia while more Heading dogs have hip luxation. However, the study recorded only 23 cases of hip dysplasia and 31 cases of traumatic injury to the hip in 2,214 clinic presentations. In TeamMate, prevalence of hip abnormalities on enrolment was 14% (11). The differences seen between these studies can probably be explained by differences in study design, with Hughes possibly recruiting dog owners that were concerned about hip disease in their teams, Cave et al. only recording dogs that were considered by their owners to be ill or injured enough to be taken to a veterinary clinic, and TeamMate recording all abnormalities irrespective of clinical significance. Based on the current data, signs of abnormalities in the hip joints may be quite common in working farm dogs. However, it is not clear whether these abnormalities are commonly associated with clinical disease. More detailed investigation is warranted into whether the recorded hip abnormalities are associated with conditions such as hind limb lameness and osteoarthritis that can impair dogs' welfare and ability to work.

A problem that occurs as a result of our data collection procedure is that we have no way of knowing whether similar abnormalities observed at different points in time are the results of the same or separate injuries or conditions. For this reason, we chose to carry out a descriptive study that focuses mainly on the first occurrence of musculoskeletal abnormalities. While we do report on second occurrence of musculoskeletal abnormalities, we did not calculate IR ratios using this data. As we did not analyze the data longitudinally, we were unable to investigate the effect of time-varying factors such as body weight, body condition, workload, and diet on the risk of dogs developing musculoskeletal abnormalities. These variables may have acted as confounders on the groups we chose to examine here. For example, differences in body weights between sexes and types of dogs may have had an impact on the incidence rates of certain types of abnormalities. Ideally, these variables should have been analyzed using a multivariable modeling approach. Future investigations should examine these risk factors, as they may be useful in determining appropriate husbandry practices necessary to minimize the risk of dogs developing musculoskeletal injury and illness. Future investigation should also examine the effect of musculoskeletal abnormalities on the lifespan and career length of working farm dogs. In combination with the work that has already been carried out, such an investigation will enable veterinarians and dog owners to make decisions about what types of musculoskeletal abnormalities are the most important to prevent and treat in order to ensure that farm dogs lead long and healthy lives.

Due to the fact that data was collected at intervals of several months, we do not have exact data on the time between enrolment and the occurrence of clinical abnormalities, and our calculation of time at risk is an approximation that assumes musculoskeletal abnormalities occurred at the halfway point between examinations. This implies that the recorded musculoskeletal abnormalities occurred evenly distributed between examinations and that they all persisted for long enough to be recorded. However, depending on the type and underlying cause of the abnormalities, they may have occurred at any time after the previous examination and persisted, or they could have occurred within days of the examination and be fully healed shortly after. Additionally, dogs may have sustained and recovered from one or more injuries in between examinations. These injuries would not have been recorded in our data at all. Assuming that recorded abnormalities in our dataset are evenly distributed could therefore be misleading, and we may also have missed a considerable number of less serious injuries. Injuries with a lower or shorter-term impact than those recorded here should not be discounted from a welfare perspective, especially if they are numerous and/or repetitive. Additionally, such injuries could have long-term consequences if they are repetitive and/or cause changes in tissues or joints. However, the abnormalities that we have reported on in this study, while possibly incomplete, still provide information about the types of injuries that occur and could be used to inform decisions around management and veterinary treatment of working farm dogs.

Another potential weakness of the TeamMate study is the reliance on veterinarians' examination notes to code clinical abnormalities. Several veterinarians participated in data collection, and different veterinarians sometimes examined the same dog at different points in time. This created a possibility that different individuals assessed and described similar or identical abnormalities in different ways. However, in order to minimize bias in the data, veterinarians were given training in how to carry out examinations in a standardized way and were asked to describe physical signs rather than to give overall diagnoses. While differences in data collected by different veterinarians are impossible to rule out, we have worked to minimize the risk of bias through our data collection, coding, and data entry procedures.

While there are several weaknesses that limit our ability to draw conclusions from the current study, this is the first time the incidence of musculoskeletal abnormalities has been investigated in working farm dogs. It is our hope that the study will form the basis for future investigation that can help improve the health and welfare of these hard-working dogs.

## Data Availability Statement

The datasets generated for this study are available on request to the corresponding author.

## Ethics Statement

The animal study was reviewed and approved by Massey University Animal Ethics Committee (protocols 15/26 and 18/53). All dog owners have given oral consent to their dogs being included in the study. Written consent is not a requirement in New Zealand and there are many cases in which projects will be approved without written consent. In this survey verbal consent was considered both acceptable and appropriate: (1) the dog owners had to agree to allow the veterinarian to visit the property, (2) when the veterinarian arrived the dog owners had to consent to them being there, and (3) the owner had to provide the dog to the veterinarian for examination. Further, at each round of data collection dog owners were free to withdraw. Several did withdraw from the study and others did not return phone calls. In terms of the actual process of ethical approval, when the proposal was sent to the Massey University Animal Ethics Committee the method of gaining consent was not included and the Committee did not require the inclusion of this prior to approval.

## Author Contributions

NC and LL were major contributors to the design of the study. LL and HW organized and contributed to data collection. KEI analyzed the data and drafted the document. NJC, EJN, and NC provided advice on analysis and interpretation of results. All authors provided revisions to the text. All authors read and approved the final manuscript.

## Conflict of Interest

The authors declare that the research was conducted in the absence of any commercial or financial relationships that could be construed as a potential conflict of interest.
